# Comparison of Oxidative Stress and Inflammation Induced by Different Intravenous Iron Sucrose Similar Preparations in a Rat Model

**DOI:** 10.2174/187152812798889358

**Published:** 2012-02

**Authors:** Jorge Eduardo Toblli, Gabriel Cao, Leda Oliveri, Margarita Angerosa

**Affiliations:** Laboratory of Experimental Medicine, Hospital Alemán, School of Medicine, University of Buenos Aires, Av. Pueyrredon 1640, (1118) Buenos Aires, Argentina

**Keywords:** Anemia, inflammation, iron deficiency, iron sucrose, iron sucrose similar, oxidative stress.

## Abstract

Iron sucrose originator (IS_ORIG_) has been used to treat iron deficiency and iron deficiency anemia for decades. Iron sucrose similars (ISSs) have recently entered the market. In this non-clinical study of non-anemic rats, five doses (40 mg iron/kg body weight) of six ISSs marketed in Asian countries, IS_ORIG_ or saline solution (control) were administered intravenously over four weeks to compare their toxicologic effects. Vasodilatory effects, impaired renal function and hepatic damage were only observed in the ISS groups. Significantly elevated serum iron and transferrin saturation levels were observed in the ISS groups suggesting a higher release of iron resulting in higher amounts of non-transferrin bound (free) iron compared to IS_ORIG_. This might explain the elevated oxidative stress and increased levels of inflammatory markers and antioxidant enzymes in the liver, heart and kidneys of ISS-treated animals. Physico-chemical analyses showed that the molecular structure of most of the ISSs differed greatly from that of the IS_ORIG_. These differences may be responsible for the organ damage and oxidative stress observed in the ISS groups. Significant differences were also found between different lots of a single ISS product. In contrast, polarographic analyses of three different IS_ORIG_ lots were identical, indicating that the molecular structure and thus the manufacturing process for IS_ORIG_ is highly consistent. Data from this study suggest that ISSs and IS_ORIG_ differ significantly. Therefore, before widespread use of these products it would be prudent to evaluate additional non-clinical and/or clinical data proving the safety, therapeutic equivalence and interchangeability of ISSs with IS_ORIG_.

## INTRODUCTION

Iron deficiency (ID) is one of the world’s most prevalent nutrient deficiencies [1]. It has various causes, such as increased iron demands due to blood loss, growth, pregnancy, inadequate dietary intake due to poor nutrition, and inadequate gastrointestinal absorption due to malabsorption or interference with drugs and food components. Because iron-containing enzymes are essential for all major metabolic processes, ID can lead to inadequate synthesis of these essential enzymes and thus to deleterious effects on cells and tissues. Untreated ID can lead to iron deficiency anemia (IDA), a condition in which the number of red blood cells (RBCs), the hemoglobin (Hb) level, and the volume of packed RBCs in the blood are below the normal values [2, 3]. IDA is an additional burden in numerous disease states including chronic heart failure (CHF) , inflammatory bowel disease and chronic kidney disease (CKD) [4-9].

Iron supplementation is often necessary to manage ID with or without anemia. The key goal is to replenish body iron stores and, if present, correct the Hb deficit [2]. Although oral iron administration is the least expensive form of iron therapy [5], the intravenous (i.v.) route of iron administration has become more favored in recent years in many clinical settings due to the gastrointestinal intolerance, low iron delivery rates, limited absorption and prolonged iron store repletion times associated with oral iron supplements [10]. The iron preparations for i.v. administration that have been available already for several years include iron sucrose, sodium ferric gluconate, and low- and high-molecular weight iron dextran. Recently, preparations that allow rapid administration of high doses of iron, such as ferric carboxymaltose (available in various countries) and ferumoxytol (available in the USA), have been marketed worldwide. All iron complexes for i.v. administration consist of a polynuclear iron(III)-oxyhydroxide core shielded by a carbohydrate shell [11, 12]. However, they vary widely in their physico-chemical properties, pharmacological activity, and side effects [11, 13, 14].

Despite the rapid recovery of the ID status in response to i.v. iron administration, there are potential risks associated with i.v. iron. Due to the risk of life-threatening/serious anaphylactic reactions associated with i.v. iron dextran, in particular high-molecular weight iron dextran, this form of iron therapy is not generally recommended [15-17]. Moreover, the more labile, low molecular weight compounds, such as ferric gluconate, release larger amounts of iron into the circulation saturating transferrin and generating non-transferrin bound iron (NTBI) [14, 18]. NTBI is taken up unspecifically by the liver, endocrine tissue and heart where it may catalyze a number of reactions that lead to oxidative stress and tissue damage [19, 20]. The extent of iron release into the circulation determines the maximum allowed single dose of an i.v. iron preparation to minimize the amount of NTBI [21]. More stable iron complexes can be administered in higher doses.

Iron sucrose is an intravenous iron preparation that contains no dextran or its derivatives, and the associated incidence of allergic side effects has been shown to be rare [11, 22]. The iron sucrose originator (IS_ORIG, _iron sucrose preparation, Venofer^®^, Vifor International, Switzerland) complex has been in clinical use for decades for the treatment of ID and IDA in a variety of clinical conditions [2]. In recent years, a number of new iron sucrose similar (ISS) preparations have entered the market. Their structures differ from that of the IS_ORIG_ complex due to different manufacturing processes [23]. These subtle structural differences may present a significant risk, raising potential concerns about the safety and efficacy of ISS preparations in clinical use. Non-clinical studies have indicated hemodynamic and functional differences between the IS_ORIG_ and ISS preparations and, in particular, a higher potential of ISSs to induce oxidative stress in kidneys, liver and heart [24, 25]. Additionally, in a recently published observational clinical study, switching the i.v. iron treatment of hemodialysis patients from IS_ORIG_ to an ISS led to a significant decrease in Hb levels and iron indices suggesting that the studied ISS preparation may not be therapeutically equivalent to IS_ORIG_ [26].

We previously established a non-clinical model that allows distinguishing between the potential of ISS preparations to induce oxidative stress in the liver, heart and kidneys [24, 25]. In the present study, we used this same model to compare the properties of six ISS preparations in the Asian market with those of the IS_ORIG_ complex.

## METHODS

### Physico-Chemical Analysis

Physico-chemical analyses were performed by the Quality Control Laboratory of Vifor (International) Ltd. (St. Gallen, Switzerland) on the samples of the ISS preparations. Molecular weight distribution was measured by gel permeation chromatography as described previously [11, 27]. The Fe(III)/Fe(II) reduction potentials were measured by polarography as described previously [11]. The turbidity point, pH and titratable alkalinity were assessed by methods described previously [27, 28].

### Animals and Treatments

All experiments were approved by the Hospital Alemán Animal Care and the Teaching and Research Committee, and performed in accordance with the *NIH Guide for the Care and Use of Laboratory Animals.* Forty male and forty female 2-month-old non-anemic Sprague-Dawley rats (Laboratory of Experimental Medicine, Hospital Alemán, Buenos Aires, Argentina) weighing 200-220 g were randomized into eight groups (*n *= 10/group) with an equal male-female distribution. The control group received isotonic saline solution and the IS_ORIG_ group received iron sucrose [Venofer^®^, LOT 517100, Vifor (International), Switzerland]. The ISS groups received either ISS_FERP_ (Ferplex^®^ SS, LOT H3797, Himont Pharmaceuticals, Lahore, Pakistan), ISS_FERI_ (Ferijet, LOT 024-Y, Akson Pharmaceuticals, Azad Kashmir, Pakistan), ISS_FERO_ (Ferosoft^®^ S, LOT 6016, Hilton Pharma Ltd., Karachi, Pakistan), ISS_ENCI_ (Encifer^®^, Emcure Pharmaceuticals Ltd., Pune, India), ISS_BACK_ (Fe-Back, Nang-Kuang Pharmaceutical Co. Ltd., Tainan Hsien, Taiwan) or ISS_LIB_ (Fe-Lib, LOT A5015, Advanced International Pharmaceutical Nanotech Inc., Taiwan).

Rats were housed in metabolic cages in a temperature-controlled room (22 ± 2 °C) subject to 12 h light/dark cycles (07.00-19.00). All animals had free access to tap water and were fed standard rat chow (16-18% protein, Cooperación, Argentina) *ad libitum* throughout the study. Rats received a single i.v. dose by tail vein injection of the corresponding iron compound (40 mg/kg body weight) or control solution (equivalent volume) at the same time every 7 days for 4 weeks (a total of five administrations on days 0, 7, 14, 21 and 28). Treatment doses were adjusted each week according to the body weight of each animal.

Blood samples were obtained for biochemical assessment of Hb, serum iron and transferrin saturation (TSAT) 24 h after an i.v. iron treatment on days 1, 8, 15, 22 and 29. Urine was collected in each group for 24 h after each i.v. injection with methods described previously [29]. The rats were sacrificed on day 29 by subtotal exsanguination under anesthesia (sodium thiopental, 40 mg/kg body weight, intraperitoneal) according to institutional guidelines for animal care and use. Previously, blood samples were obtained for biochemistry determination. The liver, heart and kidneys of each rat were perfused with ice cold saline solution through the abdominal aorta until they were free of blood and then removed for oxidative stress evaluation, microscopy and immunohisto-chemical study.

### Blood Pressure Measurement

Systolic blood pressure (SBP) was measured by tail-cuff plethysmography at baseline (day 0) and 24 h after each i.v. iron administration (days 1, 8, 15, 22, and 29). Cuff pressure was determined by a Pneumatic Pulse Transducer using a Programmed electro-sphygmomanometer PE-300 (Narco Bio-Systems, Austin, Texas, USA); rats were restrained in a plastic chamber without anesthesia. Pulses were recorded on a Physiograph MK-IIIS (Narco Bio-Systems, Austin, Texas, USA) and a minimum of three measurements were taken at each session. The SBP was calculated as an average of the three readings [29, 30].

### Biochemical Procedures

Blood samples were collected from the tail vein in capillary tubes for biochemistry determination after 14 h of fasting. Hb concentration was determined by SYSMEX XT 1800i (Roche Diagnostic GmbH, Mannheim, Germany). Serum iron was determined by radial immunodiffusion (Diffu-Plate, Biocientifica S.A., Buenos Aires, Argentina) and TSAT was obtained using traditional chemical methods. Liver enzymes, including aspartate aminotransferase (AST), alanine aminotransferase (ALT) and alkaline phosphatase (ALP), were assessed in the blood samples by colorimetric and ultraviolet (UV) methods using an Autoanalyzer Modular P800 with corresponding reagents (Roche Diagnostic GmbH, Mannheim, Germany). Aliquots of urine were assessed for creatinine with the enzymatic UV method (Randox Laboratories Ltd., Crumlin, Northern Ireland). Creatinine clearance (CrCl) was determined by the standard formula, and proteinuria was determined using the sulfosalicylic acid method.

### Evaluation of Oxidative Stress Parameters in Liver, Heart and Kidney

Samples of the whole liver, heart and kidney were homogenized (1:3, w:v) in ice cold 0.25 M sucrose solution. Glutathione (GSH) levels were determined in the 10,000 × g supernatant by methods described previously [31, 32]. Further samples of the corresponding perfused tissues were homogenized (1:10, w:v) in 0.05 M sodium phosphate buffer (pH 7.4) and used for the determination of malondialdehyde to evaluate lipid peroxidation by thiobarbituric acid reactive species (TBARS). The remaining homogenate was centrifuged at 4 °C for 15 min at 9,500 × g and the supernatant was used to measure catalase activity. The remaining tissue samples were homogenized (1:3, w/v) in ice cold sucrose solution (0.25 M). The supernatant obtained after centrifugation at 105,000 × g for 90 min was used to measure Cu, Zn superoxide dismutase (Cu, Zn-SOD) and glutathione peroxidase (GPx) activity. Enzyme units (U) were defined previously [14]. Specific activity was expressed as U/mg protein.

### Light Microscopy and Immunohistochemical Study

Decapsulated kidney, liver and heart samples were cut longitudinally, fixed in phosphate-buffered 10% formaldehyde (pH 7.2) and embedded in paraffin. Three-micron sections were cut and stained. All observations were made with a light microscope Nikon E400 (Nikon Instrument Group, Melville, New York. USA) [24, 33, 34].

Immunolabeling of specimens was carried out using a modified avidin-biotin-peroxidase technique (Vectastain ABC kit, Universal Elite, Vector Laboratories, CA, USA) as described previously [14]. Tissue ferritin was quantified with antiferritin monoclonal antibody (Biogen, San Román, California, USA). Pro-inflammatory markers were quantified with monoclonal antibodies against rat tumor necrosis factor-alpha (TNF-α) (R&D Systems, Minneapolis, MN, USA) and interleukin-6 (IL6) (Santa Cruz Biotechnology, Santa Cruz, CA, USA) at dilutions of 1:50 and 1:100, respectively (PBS diluting agent) [29, 35].

### Morphometric Analysis

Histological sections were studied in each animal with an image analyzer (Image-Pro Plus version 4 for Windows, Media Cybernetics LP, Silver Spring, MD, USA) as described previously [14]. 

### Statistical Methods

Values are expressed as mean ± SD. All statistical analyses were performed using absolute values and processed through GraphPad Prism version 5.01 for Windows (GraphPad Software, Inc. San Diego, CA, USA). For parameters with a Gaussian distribution, comparisons among groups were performed using analysis of variance (ANOVA) and for parameters with a non-Gaussian distribution using Kruskal-Wallis test (non-parametric ANOVA) and Dunn’s multiple comparison test. A value of p<0.05 was considered significant.

## RESULTS

### Physico-Chemical Analyses

The physico-chemical analyses of the studied ISS preparations showed clearly that, except for ISS_FERP_, these products do not comply with the specifications of IS_ORIG_ [27]. All ISSs, except for ISS_FERP_, had lower titratable alkalinity and out of range turbidity point (Table **1**). In addition, ISS_FERI_, ISS_BACK_, and ISS_LIB_ had a very high molecular weight. ISS_FERI _and ISS_FERO_ also had a lower pH than IS_ORIG_. Interestingly, great variations in some of the physico-chemical parameters were also observed between the different lots of the two ISSs (ISS_ENCI_ and ISS_BACK_) that were analyzed.

**Fig. (1):**
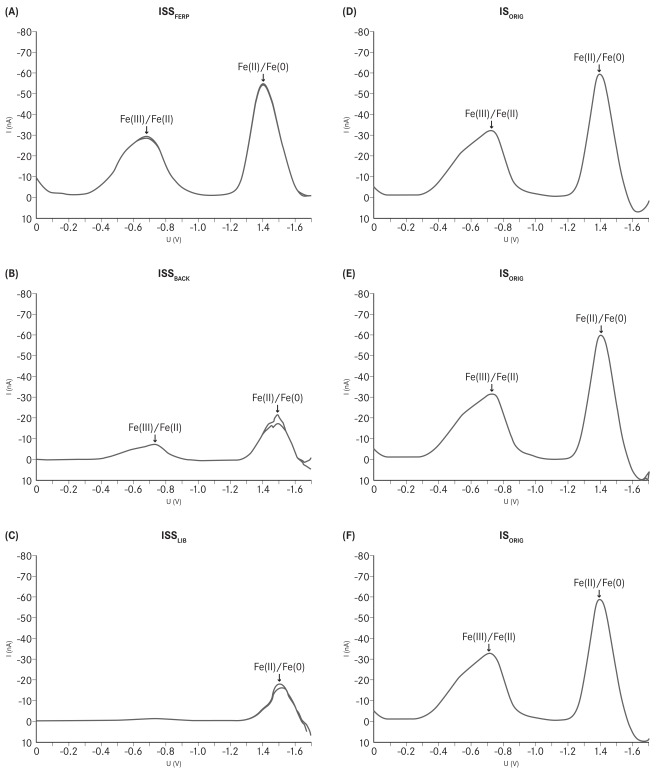
Polarograms of (**A**) ISS_FERP_, (**B**) ISS_BACK_ (Lot 5064), (**C**) ISS_LIB_, and (**D**-**F**) three randomly chosen lots of IS_ORIG_. (Studies performed by the Quality Control Laboratory of Vifor (International) Ltd., St. Gallen, Switzerland.)

The reduction potentials were determined by polarography and are measured vs. Ag/AgCl 3M KCl, if not otherwise specified. The iron(III)/iron(II) reduction potentials varied considerably among the different investigated ISSs. The reduction potential of ISS_FERP_ was the only one of the studied ISSs that complied with the reference value for iron sucrose and also the shape of its polarogram was found to be identical to that of IS_ORIG_ (Fig. **1**). The reduction potentials of ISS_BACK_ and ISS_LIB_ were not determinable due to low solubility of these compounds. In addition, the shapes of the polarograms for these ISSs were different from that of IS_ORIG_, confirming the dissimilarities between the structures of these ISSs and that of the IS_ORIG_ complex (Fig. **1**). The reduction potentials of ISS_FERI_, ISS_FERO_ and ISS_ENCI_ did not match the specifications for iron sucrose (-750 ± 50 mV) [27]. The polarogram of ISS_FERI_ revealed two distinct Fe(III)/Fe(II) transitions at -495 and -765 mV. ISS_FERO_ had a reduction potential of -566 mV, whereas ISS_ENCI_ had different values depending on the analyzed lot (-565 and -534 mV for lot LHA04003 and LHA05005, respectively). In contrast, polarograms of three randomly chosen lots of IS_ORIG_ had an identical shape and complied with the reference specification, reflecting the high consistency of the manufacturing process and thus the structure of the IS_ORIG_ complex (Fig. **1**).

### Non-Clinical Study

Throughout the non-clinical study, no significant differences were observed in Hb concentrations between the i.v. iron-treated and control groups, as expected for non-anemic rats (Table **2**). Biochemical analysis revealed that serum iron concentration and TSAT levels were significantly elevated (p<0.01) in animals from the ISS groups and the IS_ORIG_ group compared to control group on days 1, 8 and 29 (Table **2**). The IS_ORIG_ group showed significantly lower values (p<0.01) in both serum iron concentration and percentage TSAT compared to the ISS groups on days 1, 8 and 29.

All ISS groups (ISS_FERP_, ISS_FERI_, ISS_FERO_, ISS_ENCI_, ISS_BACK_, ISS_LIB_) presented a significant decrease in SBP throughout the study compared to the control and IS_ORIG_ groups (p<0.01) (Table **3**); there were no significant differences between the ISS groups themselves. 

**Fig. (2):**
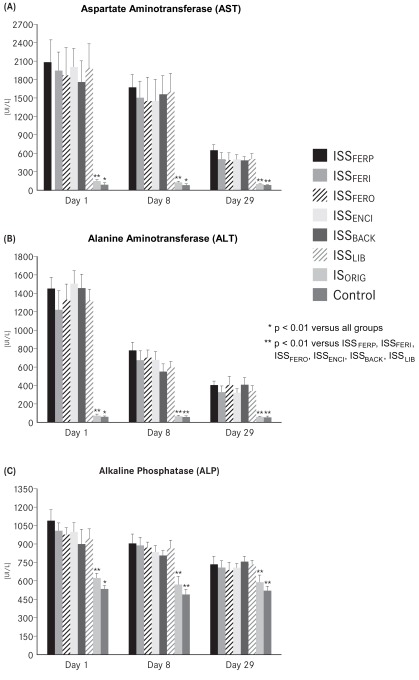
(**A**) Aspartate aminotransferase (AST), (**B**) alanine aminotransferase (ALT) and (**C**) alkaline phosphatase (ALP) after weekly i.v. administration (40 mg iron /kg body weight or equivalent volume) in the IS_ORIG_, ISS and control groups over a 4- week period.

CrCl was significantly reduced in rats from all of the ISS groups on days 1, 8 and 29 compared with the IS_ORIG_ and control groups (p<0.01) (Table **4**), whereas the values between the IS_ORIG_ and control group did not differ significantly. All ISS groups showed significant proteinuria on days 1, 8 and 29 (p<0.01) compared with the IS_ORIG_ and control groups, which showed no differences throughout the study (Table **4**). Assessment of liver function showed that AST, ALT and ALP levels were significantly increased (p<0.01) in all ISS groups on days 1, 8 and 29 compared with IS_ORIG_ and control groups (Fig. **2A-C**). Significant differences, although modest (p<0.05), were observed between the IS_ORIG_ group and the control group on days 1 and 8. However, there was no marked difference between these groups on day 29.

**Fig. (3):**
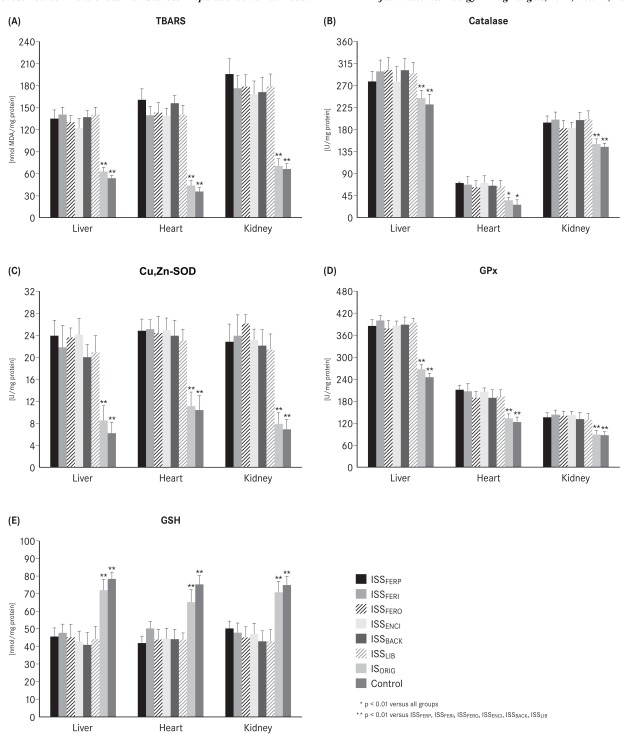
(**A**) Thiobarbituric acid reactive species (TBARS), (**B**) catalase, (**C**) Cu,Zn-superoxide dismutase (Cu,Zn-SOD), (**D**) glutathione peroxidase (GPx), (**E**) glutathione (GSH) in liver, heart and kidney homogenates after i.v. administration (40 mg iron /kg body weight or equivalent volume) in the IS_ORIG_, ISS and control groups on day 29.

The liver, heart and kidney tissues of all the ISS groups showed a significant increase (p<0.01) in malondialdehyde (TBARS), catalase, GPx, and Cu, Zn-SOD levels and a significant decrease (p<0.01) in GSH level when compared with the IS_ORIG_ and control groups on day 29 (Fig. **3A**-E). The IS_ORIG_ group showed only modest non-significant increases in the levels of malondialdehyde, catalase, GPx, and Cu, Zn-SOD in the liver and a modest non-significant decrease in the GSH levels in the liver, heart and kidneys compared to the control group on day 29. Other oxidative stress parameters did not differ between the IS_ORIG_ group and the control group in heart or kidneys at the end of the study.

On day 29, microscopy studies of the liver showed significantly more (p<0.01) positive staining for iron (Prussian blue) in the Kupffer cells, sinusoidal epithelial cells and hepatocytes in all ISS groups compared to the IS_ORIG_ group. The IS_ORIG_ group had iron deposits only in the Kupffer cells and displayed a significantly (p<0.01) greater area for ferritin staining in the liver compared to the ISS and control groups (Table **5**).

On day 29, cardiomyocytes of all ISS groups showed a significantly larger (p<0.01) area for iron staining (Prussian blue) compared to that of the cardiomyocytes of the IS_ORIG_ and control groups. Only small ferritin deposits were observed in all of the ISS groups, whereas ferritin deposits were significantly larger (p<0.01) in the IS_ORIG_ group (Table **5**).

A significant (p<0.01) positive staining for iron (Prussian blue) was detected in the proximal tubular epithelial cells of all the ISS groups compared with the IS_ORIG_ and control groups on day 29. The IS_ORIG_ group showed a larger area for ferritin deposits in the proximal tubular epithelial cells compared to all of the ISS and control groups (Table **5**).

**Fig. (4):**
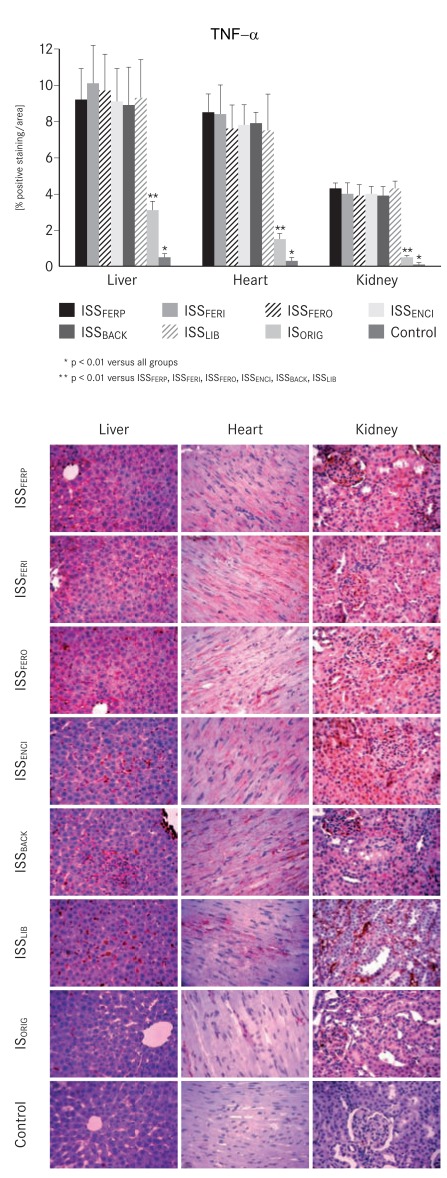
Bar chart and corresponding micrographs showing tumor necrosis factor-alpha (TNF-α) immunostaining in liver, heart and kidney samples taken from the IS_ORIG_, ISS and control groups on day 29.

**Fig. (5):**
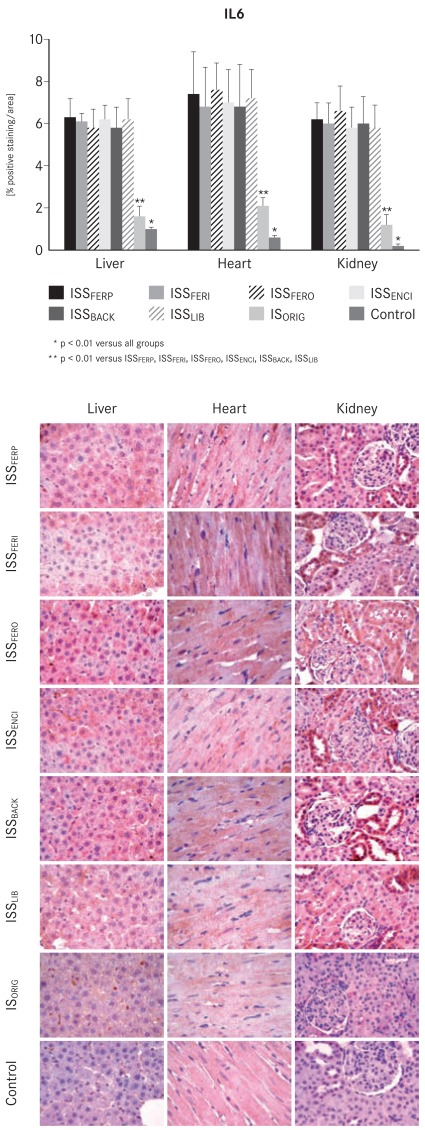
Bar chart and corresponding micrographs showing interleukin-6 (IL6) immunostaining in liver, heart and kidney samples taken from the IS_ORIG_, ISS and control groups on day 29.

Upon completion of the experiments, the levels of both inflammatory markers TNF-α and IL6 were significantly increased (p<0.01) in the liver, heart and kidney samples of the ISS groups compared with the IS_ORIG_ and control groups (Figs. **4** and **5**).

## DISCUSSION

The iron sucrose originator complex has been in clinical use for decades representing a good therapeutic option for correction of ID and IDA in more than 80 countries [2]. Together with erythropoiesis-stimulating agents it has become an integral part of IDA management associated with various clinical conditions. Clinical experience with IS_ORIG_ is based on patient exposure of more than 12 million patient years (as of 30 June 2011). IS_ORIG_ is regarded as a product with a good clinical profile [36, 37], particularly as it carries a low potential risk of hypersensitivity reactions [38].

Iron sucrose is a complex made of a polynuclear iron(III)-oxyhydroxide core stabilized by a sucrose ligand [39]. As such, it is a polymeric compound which belongs to the class of non-biological complex drugs and therefore is very different from conventional small molecule active pharmaceutical ingredients (APIs). Polymeric compounds are composed of a large number of molecules of slightly different molecular weights and are characterized by a distinct molecular weight distribution. Thus, characterization of polymeric compounds requires a set of analytical methods different from those used for conventional APIs. Due to the complexity of the process to synthesize iron sucrose, the manufacturing process largely defines the quality of the product [23, 39].

Recently, a number of ISS preparations have entered the market [23-25]. However, little is known about their safety profile as non-clinical or clinical data are not largely available. In this study, the toxicologic effects of six ISS preparations marketed in Asian countries (ISS_FERP_, ISS_FERI_, ISS_FERO_, ISS_ENCI_, ISS_BACK_, ISS_LIB_) on hemodynamic and oxidative stress parameters, inflammatory markers, tissue histologies and biochemical processes were evaluated versus IS_ORIG_ and control.

Similar to the results of the current study, our previous studies showed that the ISSs we investigated differed from IS_ORIG_ in terms of their stability and molecular structure [24, 25]. These structural differences are indicative of differences in the manufacturing process [23] and might be responsible for the negative effects of various ISS preparations that we have observed in our non-clinical studies [24, 25]. Interestingly, these subtle structural differences can escape physico-chemical characterization [23]. Recently, Meier et al. published a study in which they performed a toxicological characterization of a new ISS using a simplified version of the rat model used in the present study (shortened to an eight-day protocol). In contrast to the results presented here, their study found that the ISS had physico-chemical and toxicological properties comparable to those of IS_ORIG_ [40]. However, in a previous non-clinical study, we identified an ISS that had physico-chemical properties in compliance with the USP values for iron sucrose, but nevertheless induced a higher degree of oxidative stress and tissue damage than IS_ORIG_ [41]. Thus, even full compliance with USP-specified physico-chemical parameters does not ensure a complete characterization of an iron sucrose complex. Moreover, an observational clinical study in hemodialysis patients recently demonstrated that switching from IS_ORIG_ to an ISS led to a significant decrease in Hb levels and iron indices [26]. These findings highlight the increased need for appropriate clinical and non-clinical studies to determine the toxicological and clinical profiles of these ISS products prior to their approval [23, 42]. Consistent with this recommendation, the European Medicines Agency (EMA) recently recommended that non-clinical comparative assessments of target tissue concentrations should be performed in support of the evaluation of copies of intravenous nanoparticle iron medicinal products compared to the originator. In making their recommendation, the EMA recognized that assessments in humans based on plasma concentrations alone may not be sufficient to ensure a comparable safety and efficacy profile of these products [42].

In the present study, the results of the physico-chemical analysis of the investigated ISSs showed that only one of them, namely ISS_FERP_, complied with the USP values for iron sucrose. Within the other ISSs, significant fluctuations in the physico-chemical parameters from the reference values for iron sucrose were evident. In addition, differences in the physico-chemical parameters were also seen between the different lots of ISS_ENCI_ and ISS_BACK_. Clearly, the manufacturing processes of these two ISSs are not well controlled and thus lead to products with variable structure, stability and quality. In contrast, the manufacturing process of IS_ORIG_ is highly consistent, as shown by the identical polarograms of three randomly chosen lots. The polarographic analysis does not only indicate the potential of a complex to undergo redox cycling (reduction potential), but it is also considered as a fingerprint for the structure of the complex. Lack of physico-chemical identity with the originator demonstrates that ISS_FERI_, ISS_FERO_, ISS_ENCI_, ISS_BACK_, and ISS_LIB_ do not possess pharmaceutical equivalence to IS_ORIG_.

According to the non-clinical analysis, initial observations of the effects of the ISS compounds on SBP revealed marked hypotension. This was evident throughout the 4-week study in all ISS-treated animals, whereas SBP in the IS_ORIG_ and control groups was not affected. The vasodilatory effects associated with ISS administration highlight a potentially significant risk factor for translation to clinical practice and are consistent with the hypotensive effects reported in animals receiving ISS preparations in previous studies [24, 25].

Renal function, explored by CrCl, was not affected in rats treated with IS_ORIG_, but it was significantly reduced in the ISS groups. The reduced CrCl was accompanied by a marked proteinuria, indicating not only impaired renal function but also some disturbance in glomerular epithelial cells (podocytes) and tubular epithelial cells, in ISS-treated animals. As in our previous studies [24, 25], these results suggest that ISS preparations have more deleterious effects on the kidneys compared to the IS_ORIG_ compound. Also parallel to our previous studies, ISS induced hepatic damage was indicated by elevated levels of liver enzymes (ALT, AST and ALP) in the ISS groups compared to the IS_ORIG_ and control groups throughout the study [24, 25, 33]. The hepatic injury associated with elevated levels of liver enzymes in the blood may raise concerns regarding the use of ISS compounds in the clinical setting.

The stability of an iron complex depends on the exact structure of the complex and, in particular, on the interaction between the polynuclear iron(III)-hydroxide core and the surrounding carbohydrate [11]. As expected, in this study, all i.v. iron treated animals showed a significant increase in serum iron and TSAT levels. However, the administration of the ISS preparations resulted in significantly higher serum iron and TSAT levels than administration of the IS_ORIG_. Serum iron reflects the balance of iron flow in and out of the plasma pool [2, 43]. Therefore, higher serum iron levels induced by the ISS preparations suggest a quicker release of iron from these carbohydrate complexes causing transfer mechanisms of iron to be saturated, which leads to the generation of non-transferrin bound iron (NTBI). The more labile compounds release iron more quickly and to a greater extent, saturating transferrin, generating higher amounts of NTBI, and possibly causing oxidative stress, endothelial damage, inflammation and hemodynamic alterations [44].

NTBI is taken up unspecifically by the liver, endocrine tissue and heart where it may further catalyze reactions leading to oxidative stress and tissue damage [19, 20]. Therefore, it is not surprising that the markers for oxidative stress and the levels of antioxidant enzymes were significantly increased in the liver, heart and kidney tissues in all ISS-treated animals compared to the IS_ORIG_-treated animals and the control group throughout the study. Furthermore, oxidative stress can also increase the risk of endothelial damage and inflammation [44, 45]. The levels of both pro-inflammatory markers TNF-α and IL6 were substantially higher in the ISS groups relative to IS ORIG and control groups at the end of the study. These results further confirm that IS_ORIG_ has a lower potential to induce oxidative stress and inflammation in this non-clinical model. From a clinical point of view, these findings may be important since i.v. iron therapy is often used to treat ID or IDA in CKD, CHF and cancer patients, who are already subject to increased levels of oxidative stress. This is particularly relevant for hemodialysis patients, who necessitate frequent administration of i.v. iron. 

*In vitro* experiments have also shown differences among commonly used i.v. iron preparations. In particular, there is accumulating evidence that depending on the stability and the redox properties of the iron complex, iron preparations can influence cytokine activation, reactive oxygen species generation, and lymphocyte survival to different degrees [46-48]. 

In this study, the iron(III)/iron(II) reduction potentials of the different ISSs, except ISS_FERP_, showed great variations from the USP values for iron sucrose and that of IS_ORIG_. The reduction potentials of ISS_FERI_, ISS_FERO_ and ISS_ENCI_ were more positive than that of IS_ORIG_, suggesting that these ISS preparations may undergo redox cycling under physiological conditions and thus cause oxidative stress. Previous studies also showed that other ISSs with a more positive reduction potential than that of IS_ORIG_ induced oxidative stress in an analogous non-clinical model [24, 25]. Interestingly, ISS_FERI_ was found to exhibit two distinct reduction potentials whereas ISS_ENCI _had different reduction potentials depending on the analyzed lot. 

Cellular uptake, transient storage and subsequent utilization of iron are influenced in part by the type of iron preparation administered and in part by the dosage, treatment regimen and physiological status of the patient [49]. Iron from the ideal iron preparation is transiently deposited in the reticuloendothelial system and not in the parenchyma of the liver [11]. IS_ORIG_ shows exactly this pattern, with a more pronounced increase in iron and ferritin in the liver, and only a smaller increase relative to the control group in the heart and kidney. The presence of NTBI and oxidative stress in the livers of animals of the ISS groups was consistent with positive staining for iron not only in the Kupffer cells but also in the surrounding tissue. Accordingly, ferritin deposits, in particular in the liver, were reduced in the ISS groups suggesting that iron was stored in other cellular compartments rather than in the endogenous storage protein ferritin found predominantly in the liver. 

The data from this study along with the results from three additional ISSs tested in two previously published studies [24, 25] show that various ISSs have slightly different toxicological patterns. In particular, the organs analyzed (liver, heart, and kidney) were not always affected to the same extent. For instance, most ISSs from this study showed a more pronounced effect on the kidneys (as seen from the proteinuria, IL6, iron and ferritin data), whereas significant differences in liver toxicity have been observed for two ISSs tested in a previous study [24].

Despite the significant differences that have been observed between IS_ORIG_ and the investigated ISSs, the clinical relevance of the toxicological model used in this study and the significance of the results obtained require further discussion. The i.v. administration route adopted in this study is similar to clinical practice since iron sucrose can be given by both injection as well as infusion. However, the single iron doses used in the clinic are usually lower than those used in this study. Regarding oxidative stress, several clinical studies have investigated changes in biomarkers of oxidative stress after i.v. iron administration and demonstrated an inverse relation between the stability of the iron complexes and the induction of oxidative stress [46, 47, 50-52]. Accordingly, differences in the physicochemical properties and stability of ISSs compared to IS_ORIG_ as demonstrated in the present study may also be expected to affect oxidative stress levels in the clinical setting. In addition, differences observed in the present study in the distribution of iron that resulted in the deposition of iron in the parenchymal tissues instead of the reticuloendothelial system may lead to a decrease in the amount of iron that is available for effective erythropoiesis and, therefore, partly explain the recently published clinical observation of a lower efficacy of an ISS in maintaining Hb and iron status parameters in hemodialysis patients after the switch from IS_ORIG_ [26]. 

Overall, the significant differences observed for most of the ISSs compared to the IS_ORIG_, raise potential safety concerns on the interchangeabilty of these i.v. iron preparations and suggest that careful non-clinical and clinical evaluations of the safety and efficacy of ISSs should be performed before exposing vulnerable patients to potential additional sources of oxidative stress and inflammation. 

## CONCLUSION

The results from this study support those of previous comparative non-clinical ISS studies highlighting the extent and severity of the effects that variations in manufacturing procedures for iron sucrose preparations can cause. The physico-chemical characterization demonstrated that five of the six studied ISS preparations do not comply with the specifications of the USP Monograph for iron sucrose injection. Differences of variable degree in the molecular structures of these compounds arise from variations in the manufacturing process and may be responsible for the toxic effects that were observed in this non-clinical model. In contrast, polarographic analyses of three different IS_ORIG_ lots were identical and thus confirmed the high degree of consistency in the manufacturing process of IS_ORIG_. Interestingly, ISS_FERP_, which met the physico-chemical reference values for iron sucrose, induced an elevated level of oxidative stress compared to IS_ORIG_. Thus demonstrating that similar physico-chemical properties do not ensure similar toxicological effects. The reduced ability of the investigated ISSs to provide iron in the form that can be stored in ferritin, as well as the increased iron release indicated by higher oxidative stress marker levels, led to deleterious effects on hemodynamic, functional and inflammatory responses. In conclusion, all of the studied ISS preparations marketed in Asian countries showed different toxicological effects in rats compared to the iron sucrose originator and therefore, before administering such products in the clinical setting, it is suggested that therapeutic equivalence to the originator product ought to be proved to avoid exposing patients to potentially less safe preparations.

## Figures and Tables

**Table 1.** Characteristics, pH, Titratable Alkalinity, Turbidity Point, and Molecular Weight of the Six Asian ISS Preparations Compared with the Originator Iron Sucrose (IS_ORIG_) and the Pharmacopeia (USP). (Studies Performed by the Quality Control Laboratory of Vifor (International), St. Gallen, Switzerland)

Parameter	USP^a^	IS_ORIG_	ISS_FERP_	ISS_FERI_	ISS_FERO_	ISS_ENCI_	ISS_ENCI_	ISS_BACK_	ISS_BACK_	ISS_LIB_
**Lot**	-	517100	H3797	024-T	6016	LHA04003	LHA05005	F5097	D5064	A5015
**Characteristics**	-	Dark brown, opaque aqueous solution	Complies	Complies	Complies	Complies	Complies	Complies	Complies	Complies
**pH**	10.5-11.1	10.9	10.5	*10.4*	*9.8*	10.6	10.8	10.8	10.5	10.6
**Titratable alkalinity (ml)**	0.5-0.8	0.8	0.55	*0.4*	*0.18*	*0.33*	*0.4*	0.46	0.38	*0.37*
**Turbidity point**	4.4-5.3	4.9	5.1	*9.7*	*4.2*	*5.8*	*6.4*	*N/A^b^*	*N/A^b^*	*5.33*
**Mw (Da)**	34,000-60,000	45,700	50,600	*215,000*	39,200	50,500	46,400	*293,000*	*162,000*	*246,000*
**Mn (Da)**	≥24,000	33,900	36,100	85,200	31,300	35,100	34,200	175,000	93,300	151,000
**P**	≤1.7	1.3	1.4	*2.52*	1.25	1.44	1.36	1.67	*1.74*	1.63

a [27]

b Starting solution turbid

N/A, not available; Mw (Da), weight average molecular weight in Dalton; Mn (Da), number average molecular weight in Dalton; P = ratio Mw/Mn

**Table 2.** Hemoglobin (Hb), Serum Iron and Transferrin Saturation (TSAT)

Mean ± SD	ISS_FERP _(n = 10)	ISS_FERI _(n = 10)	ISS_FERO _(n = 10)	ISS_ENCI _(n = 10)	ISS_BACK _(n = 10)	ISS_LIB _(n = 10)	IS_ORIG _(n = 10)	Control (n = 10)
**Day 1**								
**Hb (g/dL)**	15.8 ± 0.2	15.8 ± 0.1	16.0 ± 0.1	16.1 ± 0.1	15.9 ± 0.2	15.8 ± 0.2	15.9 ± 0.2	15.8 ± 0.5
**Serum iron (µg/dL)**	559.1 ± 60.2	563.2 ± 55.3	539.0 ± 59.1	542.2 ± 50.0	549.9 ± 47.1	555.1 ± 60.0	360.0 ± 42.0#	309.9 ± 15.0*
**TSAT (%)**	86.9 ± 4.3	90.0 ± 4.1	87.7 ± 5.0	86.8 ± 5.9	86.9 ± 6.0	89.2 ± 3.9	73.2 ± 6.2#	45.1 ± 3.8*
**Day 8**								
**Hb (g/dL)**	16.2 ± 0.1	16.4 ± 0.2	16.3 ± 0.2	16.1 ± 0.1	16.4 ± 0.1	16.4 ± 0.2	16.4 ± 0.2	15.9 ± 0.4
**Serum iron (µg/dL)**	489.0 ± 52.0	500.0 ± 66.0	499.0 ± 58.0	488.3 ± 45.0	500.1 ± 32.0	479.9 ± 52.0	398.0 ± 33.0#	318.0 ± 18.0*
**TSAT (%)**	85.9 ± 3.0	87.0 ± 5.2	89.9 ± 6.1	89.4 ± 6.0	88.3 ± 4.9	88.2 ± 5.2	69.9 ± 5.5#	48.3 ± 5.9*
**Day 29**								
**Hb (g/dL)**	16.4 ± 0.2	16.5 ± 0.2	16.5 ± 0.1	16.5 ± 0.2	16.5 ± 0.2	16.5 ± 0.1	16.5 ± 0.2	16.0 ± 0.5
**Serum iron (µg/dL)**	478.1 ± 45.2	458.9 ± 60.0	486.3 ± 43.0	453.7 ± 41.1	469.5 ± 56.2	487.9 ± 39.9	395.7 ± 21.0#	301.0 ± 10.8*
**TSAT (%)**	84.4 ± 4.2	89.9 ± 6.1	88.9 ± 5.3	89.0 ± 5.7	84.8 ± 5.1	86.6 ± 4.4	70.1 ± 4.5#	47.2 ± 4.6*

* p < 0.01 versus all groups

# p < 0.01 versus ISS_FERP_, ISS_FERI_, ISS_FERO_, ISS_ENCI_, ISS_BACK_, ISS_LIB_

**Table 3.** Systolic Blood Pressure (mean ± SD) After Weekly i.v. Administration (40 mg Iron/kg Body Weight or Equivalent Volume) in ISS, IS_ORIG_ and Control Groups Over a 4-Week Period

Day	ISS_FERP _(n = 10)	ISS_FERI _(n = 10)	ISS_FERO _(n = 10)	ISS_ENCI _(n = 10)	ISS_BACK _(n = 10)	ISS_LIB _(n = 10)	IS_ORIG _(n = 10)	Control (n = 10)
**0**	118.1 ± 2.4	119.0 ± 2.9	118.1 ± 2.4	119.0 ± 2.9	118.1 ± 2.3	119.1 ± 2.9	118.9 ± 2.2	118.9 ± 1.9
**1**	112.3 ± 1.9	111.0 ± 0.1	112.3 ± 1.9	114.1 ± 1.9	112.3 ± 1.9	112.0 ± 1.9	116.2 ± 2.0#	120.2 ± 0.5*
**8**	111.4 ± 1.7	112.0 ± 3.1	114.7 ± 1.7	113.6 ± 3.1	114.7 ± 1.7	110.1 ± 3.1	117.8 ± 2.8#	119.8 ± 2.1#
**15**	112.6 ± 1.8	111.1 ± 2.4	114.9 ± 1.8	114.1 ± 2.4	116.8 ± 1.8	114.7 ± 2.4	118.0 ± 2.2#	120.3 ± 2.1#
**22**	113.7 ± 2.0	114.2 ± 2.5	114.5 ± 2.0	112.1 ± 2.5	114.5 ± 2.0	113.9 ± 2.5	119.4 ± 1.9#	120.3 ± 2.0#
**29**	115.1 ± 1.8	114.8 ± 3.0	113.6 ± 2.5	114.7 ± 2.6	113.7 ± 2.0	113.8 ± 3.0	121.2 ± 2.6#	122.1 ± 2.4#

* p< 0.01 versus all groups

# p< 0.01 versus ISS_FERP_, ISS_FERI_, ISS_FERO_, ISS_ENCI_, ISS_BACK_, ISS_LIB_

**Table 4.** Creatinine Clearance and Proteinuria After Weekly i.v. Administration (40 mg Iron/kg Body Weight or Equivalent Volume) in the ISS, IS_ORIG_ and Control Groups Over a 4-Week Period

Day	ISS_FERP _(n = 10)	ISS_FERI _(n = 10)	ISS_FERO _(n = 10)	ISS_ENCI _(n = 10)	ISS_BACK _(n = 10)	ISS_LIB _(n = 10)	IS_ORIG _(n = 10)	Control (n = 10)
**CrCl**								
**1**	2.4 ± 0.1	2.3 ± 0.1	2.4 ± 0.2	2.1 ± 0.1	2.2 ± 0.1	2.3 ± 0.2	2.7 ± 0.1#	2.8 ± 0.1*
**8**	3.3 ± 0.1	2.2 ± 0.1	2.3 ± 0.1	2.2 ± 0.2	2.3 ± 0.2	2.3 ± 0.1	2.8 ± 0.1#	2.9 ± 0.1#
**29**	2.1 ± 0.1	2.2 ± 0.1	2.2 ± 0.1	2.3 ± 0.2	2.1 ± 0.2	2.4 ± 0.2	2.7 ± 0.1#	2.9 ± 0.1#
** Proteinuria**								
**1**	19.2 ± 5.5	22.1 ± 6.2	16.9 ± 7.1	18.5 ± 7.9	17.4 ± 5.8	19.9 ± 5.8	2.0 ± 1.3#	1.2 ± 2.0#
**8**	21.9 ± 6.6	26.3 ± 7.7	27.3 ± 9.9	33.2 ± 5.8	22.9 ± 4.9	25.1 ± 4.1	5.7 ± 2.4#	4.1 ± 2.1#
**29**	35.4 ± 7.0	31.8 ± 8.2	30.7 ± 5.9	39.4 ± 8.2	35.1 ± 5.8	29.8 ± 7.9	6.3 ± 3.0#	3.8 ± 3.0#

* p< 0.01 versus all groups

# p< 0.01 versus ISS_FERP_, ISS_FERI_, ISS_FERO_, ISS_ENCI_, ISS_BACK_, ISS_LIB_

**Table 5.** Prussian Blue Staining and Ferritin Immunostaining in the Liver, Heart and Kidney Samples of the Six Asian ISS Groups, the IS_ORIG_ Group, and the Control Group on Day 29

Mean ± SD	ISS_FERP _(n = 10)	ISS_FERI _(n = 10)	ISS_FERO _(n = 10)	ISS_ENCI _(n = 10)	ISS_BACK _(n = 10)	ISS_LIB _(n = 10)	IS_ORIG _(n = 10)	Control (n = 10)
**Prussian blue (% positive staining/area)**								
**Liver**	6.8 ± 2.0	15.1 ± 3.2	14.6 ± 2.2	14.2 ± 2.6	14.7 ± 2.8	14.9 ± 1.8	7.4 ± 2.0#	1.2 ± 0.5*
**Heart**	4.1 ± 0.7	3.8 ± 0.6	4.0 ± 0.7	3.6 ± 0.8	3.9 ± 0.7	3.7 ± 0.8	1.1 ± 0.3#	0.2 ± 0.1*
**Kidney**	7.1 ± 1.0	6.2 ± 1.2	6.6 ± 1.5	6.4 ± 1.8	6.8 ± 1.3	6.3 ± 1.4	3.1 ± 0.8#	1.3 ± 0.1*
**Ferritin (% positive staining/area)**								
**Liver**	7.3 ± 2.0	6.9 ± 1.7	6.8 ± 1.4	7.5 ± 2.0	7.5 ± 1.1	7.1 ± 2.0	15.2 ± 1.4#	2.1 ± 0.6*
**Heart**	1.8 ± 0.4	1.7 ± 0.3	1.9 ± 0.4	1.7 ± 0.5	1.4 ± 0.5	1.6 ± 0.6	3.1 ± 0.4#	0.2 ± 0.1*
**Kidney**	3.6 ± 0.5	3.0 ± 0.5	2.7 ± 0.7	3.3 ± 0.5	3.6 ± 0.3	3.4 ± 0.6	6.1 ± 0.7#	0.2 ± 0.1*

* p < 0.01 versus all groups

# p < 0.01 versus ISS_FERP_, ISS_FERI_, ISS_FERO_, ISS_ENCI_, ISS_BACK_, ISS_LIB_

## ABBREVIATIONS

ALP: = Alkaline phosphatase
ALT: = Alanine aminotransferase
AST: = Aspartate aminotransferase
CHF: = Chronic heart failure
CKD: = Chronic kidney disease
CrCl: = Creatinine clearance
Cu,Zn-SOD: = Cu,Zn superoxide dismutase
GPx: = Glutathione peroxidase
GSH: = Glutathione
Hb: = Hemoglobin
i.v.: = Intravenous
ID: = Iron deficiency
IDA: = Iron deficiency anemia
IL6: = Interleukin-6
IS_ORIG_: = Iron sucrose originator
ISS: = Iron sucrose similar
ISS_BACK_: = Iron sucrose similar Fe-Back
ISS_ENCI_: = Iron sucrose similar Encifer^®^
ISS_FERI_: = Iron sucrose similar Ferijet
ISS_FERO_: = Iron sucrose similar Ferosoft^®^-S
ISS_FERP_: = Iron sucrose similar Ferplex^®^ SS
ISS_LIB_: = Iron sucrose similar Fe-Lib
NTBI: = Non-transferrin bound iron
RBC: = Red blood cell
SBP: = Systolic blood pressure
TBARS: = Thiobarbituric acid reactive species
TNF-α: = Tumor necrosis factor-alpha
TSAT: = Transferrin saturation
USP: = The United States Pharmacopeia
